# Interplay of epilepsy and long-term potentiation: implications for memory

**DOI:** 10.3389/fnins.2024.1451740

**Published:** 2025-01-10

**Authors:** Luis A. Marin-Castañeda, Gerónimo Pacheco Aispuro, Guillermo Gonzalez-Garibay, Carlos Alejandro Martínez Zamora, Hector Romo-Parra, Moisés Rubio-Osornio, Carmen Rubio

**Affiliations:** ^1^Department of Neurophysiology, Instituto Nacional de Neurología y Neurocirugía “Manuel Velasco Suárez”, Mexico City, Mexico; ^2^Department of Neuroscience, Hospital Ángeles del Pedregal, Mexico City, Mexico; ^3^Anahuac University, Mexico City, Mexico; ^4^School of Medicine, Saint Luke, Mexico City, Mexico; ^5^Universidad Iberoamericana, Mexico City, Mexico; ^6^Department of Neurochemistry, Instituto Nacional de Neurología y Neurocirugía “Manuel Velasco Suárez”, Mexico City, Mexico

**Keywords:** long-term potentiation, epilepsy, synaptic plasticity, neuroinflammation, memory

## Abstract

The interplay between long-term potentiation (LTP) and epilepsy represents a crucial facet in understanding synaptic plasticity and memory within neuroscience. LTP, a phenomenon characterized by a sustained increase in synaptic strength, is pivotal in learning and memory processes, particularly in the hippocampus. This review delves into the intricate relationship between LTP and epilepsy, exploring how alterations in synaptic plasticity mechanisms akin to those seen in LTP contribute to the hyperexcitable state of epilepsy. This state is conceptualized as a dysregulation between LTP and LTD (Long-term depression), leading to pathologically enhanced synaptic efficacy. Additionally, the role of neuroinflammation in both LTP and epilepsy is examined, highlighting how inflammatory mediators can influence synaptic plasticity. The dual role of neuroinflammatory pathways, enhancing or inhibiting LTP, is a focal area of ongoing research. The significance of various signaling pathways, including the MAPK, mTOR, and WNT/β-catenin pathways, in the modulation of synaptic plasticity and their relevance in both LTP and epilepsy. These pathways are instrumental in memory formation, consolidation, and epileptogenesis, illustrating a complex interaction between cellular mechanisms in the nervous system. Lastly, the role of calcium signaling in the relationship between LTP and epilepsy is scrutinized. Aberrant calcium signaling in epilepsy leads to an enhanced, yet pathologically altered, LTP. This dysregulation disrupts normal neural pathways, potentially leading to cognitive dysfunction, particularly in memory encoding and retrieval. The review emphasizes the need for targeted interventions in epilepsy that address cognitive functions alongside seizure control.

## 1 Introduction

Long-term potentiation (LTP) stands as a fundamental concept in neuroscience, pivotal for understanding the mechanisms of synaptic plasticity and memory. Discovered in the early 1970s, LTP is characterized by a sustained increase in synaptic strength following a brief, high-frequency stimulation of afferent fibers (Bliss and Lomo, 1973). This phenomenon, primarily observed in the hippocampus, a brain region integral to learning and memory, has been extensively studied to unravel the cellular and molecular mechanisms underlying memory formation. LTP manifests in two phases: an initial, transient phase (early LTP) that involves post-translational modifications of proteins, and a late phase (late LTP) that requires new protein synthesis and is associated with structural changes at the synapse. Traditionally, LTP is considered a postsynaptic mechanism; however, evidence suggests it may also function as a presynaptic mechanism in specific regions, such as CA3 of the hippocampus (Yang and Calakos, 2013; El Oussini et al., 2023). The induction of LTP typically involves NMDA receptor activation, leading to calcium influx and subsequent activation of various kinases, which collectively contribute to the strengthening of synaptic connections. The study of LTP has revolutionized our understanding of how experiences can lead to lasting changes in brain function, providing a cellular correlate for learning and memory (Muller et al., 2002).

The relationship between LTP and epilepsy presents a fascinating yet complex facet of neuroscience research. Epilepsy, a disorder characterized by recurrent seizures, is thought to involve alterations in synaptic plasticity mechanisms similar to those seen in LTP. The hyperexcitable state in epilepsy can be conceptualized as an imbalance in the normal processes of synaptic strengthening (LTP) and weakening (long-term depression, LTD), leading to a pathological enhancement of synaptic efficacy (Kopec et al., 2007; Potter et al., 2010). This dysregulation may result in the formation of hyperconnected networks that are more susceptible to synchronized, seizure-like activity. Studies have shown that seizures themselves can induce LTP-like changes in the brain, suggesting a bidirectional relationship where not only does LTP contribute to the development of epilepsy, but epileptic activity can also enhance LTP mechanisms (Grosser et al., 2020).

The kindling model, initially described by Goddard et al. (1969), further illustrates the connection between epilepsy and LTP. Developed as an experimental approach to study LTP through the repetitive application of subconvulsive electrical stimuli to brain regions, kindling leads to progressive and spontaneous seizure development (Goddard et al., 1969; Faas et al., 1996). This model mirrors the synaptic strengthening seen in LTP and was foundational in establishing the link between these phenomena. The repetitive stimulation in kindling parallels the synaptic enhancement observed in LTP, suggesting shared molecular and cellular mechanisms (Roohi et al., 2021). The kindling model has thus been pivotal in demonstrating how persistent LTP-like changes can lead to the development of a hyperexcitable neuronal network, shedding light on the dual roles of synaptic plasticity in both cognitive processes and the pathogenesis of neurological disorders (Danzer et al., 2010).

The focus of this review is to explore the intricate interplay between epilepsy and LTP, with an emphasis on the intracellular signaling pathways involved. By understanding these mechanisms, we aim to identify potential targets that could modulate synaptic plasticity, thereby preventing or reducing the frequency and severity of seizures.

## 2 Neuroinflammatory pathway

Neuroinflammation, once primarily associated with neurodegenerative diseases, is now recognized as a significant factor in various neurological conditions, including epilepsy. Recent research has begun to elucidate the complex role of neuroinflammatory pathways in both LTP and epilepsy, revealing a multifaceted relationship that influences neuronal excitability and synaptic plasticity (Wang et al., 2010; Iwai et al., 2014).

Neuroinflammation involves the activation of glial cells, including microglia and astrocytes. These cells release inflammatory mediators such as cytokines and chemokines. Acute neuroinflammation can be protective, facilitating adaptive responses that support neural plasticity. However, chronic inflammation typically impairs neuronal functions and survival. Beyond their role in inflammation, astrocytes actively participate in the synaptic environment that supports LTP. They interact with neurons to regulate synaptic plasticity, not only by modulating neurotransmitter clearance and ionic balance but also through their own intracellular signaling pathways such as p38 MAPK. This astrocytic signaling influences both excitatory and inhibitory neurotransmission, crucial for the maintenance and modulation of LTP (Navarrete et al., 2019). By expressing receptors and signaling molecules involved in pathways such as p38 MAPK, astrocytes respond to synaptic activity and environmental changes, thereby directly influencing the dynamics of LTP (Arruda et al., 1998; Kim et al., 2020). It has been shown that these neuroinflammatory mediators can impact synaptic plasticity, enhancing or inhibiting LTP. Proinflammatory cytokines such as IL-1, TNF, COX-2, and HGMB significantly contribute to the physiopathology of epilepsy (Sun et al., 2024). These cytokines can be produced both centrally and peripherally and have been extensively studied as potential therapeutic targets in epilepsy treatment (Jin et al., 2019; Li et al., 2019; Wang et al., 2021). They modulate neuronal excitability and synaptic function, which can exacerbate seizure activity and contribute to the chronic nature of the disease.

Experimental findings in a study using C57BL/6 adult male mice treated acutely with different doses of lipopolysaccharide (LPS), an endotoxin, and ibuprofen (IBU), a nonselective cyclooxygenase inhibitor, illustrate the dose-dependent effects of inflammatory modulators on neural plasticity. Both LPS and IBU, at higher doses, significantly decreased the amplitude of LTP, Brain-Derived Neurotrophic Factor (BDNF) expression levels, and phosphorylation of the AMPA receptor subunit GluR1, indicating an impairment in synaptic plasticity regardless of whether the inflammatory response was upregulated or downregulated (Golia et al., 2019).

Moreover, neuroinflammation can alter synaptic plasticity through the regulation of NMDA and AMPA receptors (Postnikova et al., 2017; Sun et al., 2024). The release of proinflammatory cytokines and chemokines may influence glutamatergic pathways (Auvin et al., 2010), affecting the LTP. This suggests a complex interplay between inflammatory processes and synaptic changes in the context of epilepsy. This relationship between LTP alterations during epileptic seizures and neuroinflammation has yielded conflicting results. Neuroinflammatory pathways are implicated in various mood and behavioral disorders, more prevalent in individuals with epilepsy than in the general population (Wang et al., 2010). Studies have shown an increase in the hippocampal mRNA expression of cytokines like IL-1β, IL-6, and TNF-a during seizures, impacting learning and memory in mice (Han et al., 2016). Interestingly, LTP was enhanced by anakinra, an anti-interleukin-1 receptor antagonist used in treating rheumatoid arthritis. This suggests that specific cytokine pathways might have distinct roles in modulating synaptic plasticity and cognitive functions in epilepsy. Specifically, IL-1β, a well-known proinflammatory cytokine, has been documented to inhibit the induction phase of LTP in the hippocampus. This inhibition is mediated through its action on calcium dynamics within neurons. IL-1β modulates the influx of calcium through NMDA receptors, crucial for the induction of LTP. It also activates stress-activated protein kinases that interfere with synaptic strengthening, thus disrupting the early phase of LTP, which relies on post-translational modifications of existing synaptic proteins (Viviani et al., 2003; Tong et al., 2008).

**FIGURE 1:**
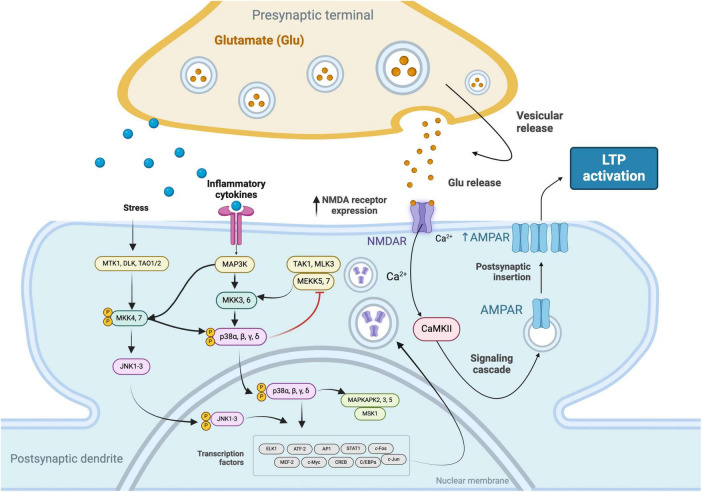
Signaling cascades that facilitate the increased expression of N-Methyl-D-Aspartate (NMDA) receptors in response to stress stimuli and inflammatory cytokines. These pathways involve the convergence of the c-Jun N-terminal Kinase (JNK) 1-3 and Mitogen-Activated Protein Kinase Kinase Kinase (MAP3K) pathways, leading to the phosphorylation and nuclear translocation of p38 MAPKs (α, β, γ, δ) and the activation of transcription factors. The subsequent upregulation of NMDA receptors enhances synaptic responsiveness to glutamate, resulting in elevated intracellular Ca^2 +^ levels. This calcium influx activates Calcium/Calmodulin-Dependent Protein Kinase II (CaMKII), initiating a signaling cascade that culminates in the increased expression of α-Amino-3-Hydroxy-5-Methyl-4-Isoxazolepropionic Acid (AMPA) receptors, thereby promoting LTP.

Conversely, TNF-α has been shown to play a dual role in the modulation of LTP. It can enhance LTP by increasing the surface expression of AMPA receptors at synapses, which is essential for the maintenance phase of LTP, characterized by sustained synaptic strengthening. This effect of TNF-α promotes the consolidation of synaptic efficacy. However, at high concentrations, TNF-α contributes to excitotoxicity and impairs LTP by overactivating glutamate receptors, thus highlighting its complex role in synaptic plasticity (Beattie et al., 2002; Stellwagen and Malenka, 2006; Figure 1).

In the broader context of neuroinflammation’s impact on synaptic plasticity, it is also important to consider the specific roles of NMDA receptor subunits in the induction of LTP. The GluN2A subunit, typically localized synaptically, is instrumental in LTP induction, whereas the GluN2B subunit, often found at extrasynaptic sites, is associated with LTP depression (Parsons and Raymond, 2014). Neuroinflammatory processes, particularly those involving cytokines like IL-1β and the activation of pathways such as PI3K/Akt, can influence the production and localization of these subunits. For instance, increased IL-1β levels might lead to a rise in GluN2B at extrasynaptic sites, potentially diminishing LTP (Li et al., 2016; Delgado et al., 2018; Cianciulli et al., 2020). This connection between neuroinflammatory signaling, receptor subunit dynamics, and synaptic plasticity underlines the intricate interplay between cellular mechanisms in epilepsy.

However, the role of neuroinflammation in LTP is not straightforward, moderate levels of inflammatory mediators might facilitate LTP, while excessive inflammation typically impairs synaptic plasticity. This dual role is a key area of ongoing research (Park et al., 2021).

The IL-1 receptor antagonists (IL-1Ra) represent a promising therapeutic target and an area ripe for future research. These antagonists can inhibit the action of IL-1β, which, in turn, may restore normal synaptic plasticity and reduce the hyperexcitability associated with seizures (Vezzani et al., 2011). By blocking IL-1β signaling, IL-1Ra has the potential to significantly impact the treatment of this condition.

## 3 MAPK pathway

**FIGURE 2:**
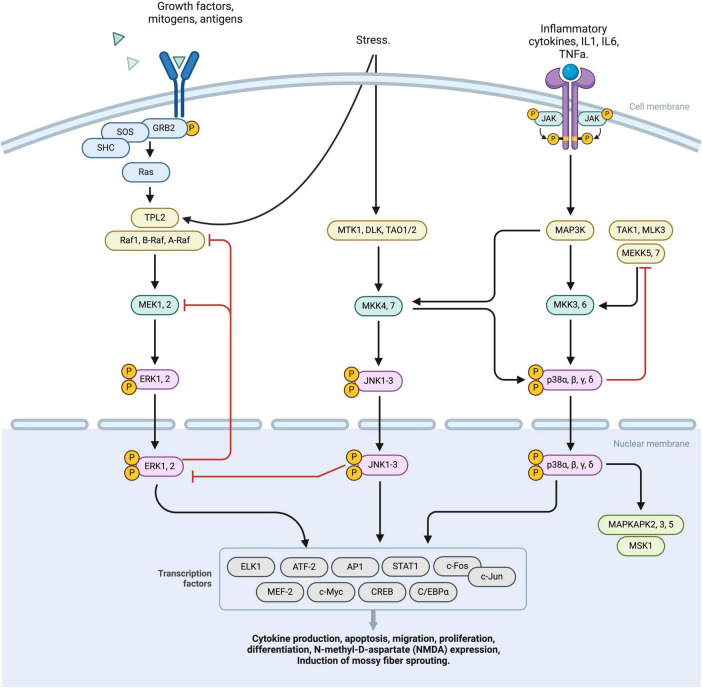
This diagram represents a schematic overview of the MAPK pathway, comprising three principal activation branches: RTK, ERK, JNK activation nodes. The binding of specific ligands to the RTK receptor initiates the phosphorylation of the RAS complex, subsequently activating the TPL2 factor. This TPL2 factor then converges with the ERK pathway, sequentially activating MEK 1,2, ERK1,2, which translocates intranuclearly and stimulates various transcriptional processes. External stress stimuli induce the activation of MTK1, DLK, TAO1/2 complexes leading to phosphorylation of JNK 1-3. JNK 1-3 operates at a nuclear level, modulating transcription factors. Furthermore, JNK receptor activation exerts downregulation effects on various factors, including MAP3k and MKK3,6. This cascade culminates in the phosphorylation of p38 α,β,γ,δ, facilitating its nuclear translocation and the regulation of multiple transcription factors. Regulatory negative feedback mechanisms interconnect the RTK, ERK, and JNK pathways, finely tuning the cellular response to MAPK pathways by either downregulating or upregulating specific cellular processes. MAPK, Mitogen-activated protein kinases; RTK, receptor tyrosine kinases; ERK, extracellular signal-regulated kinase; JNK, Janus kinases; TPL2, Tumor progression locus 2; MEK1,2, Mitogen-Activated Protein Kinase Kinase 1 and 2; ERK1,2, Extracellular Signal-Regulated Kinase 1 and 2; MTK1, MAP3K Protein Kinase 1; DLK, Dual Leucine Zipper Kinase; TAO1/2, Thousand And One Kinase 1 and 2; JNK1-3, c-Jun N-terminal Kinases 1, 2, and 3; MAP3K, Mitogen-Activated Protein Kinase Kinase Kinase; MKK3,6, Mitogen-Activated Protein Kinase Kinase 3 and 6; p38, p38 Mitogen-Activated Protein Kinase α,β,γ,δ.

The MAPK (mitogen-activated protein kinase) pathway plays a significant role in the development and progression of epilepsy. This pathway consists of three major branches: the extracellular signal-regulated kinase (ERK) pathway, the p38 pathway, and the C-Jun N-terminal kinases (JNK) pathway. These pathways are important for cell signal transduction and respond to various stimuli such as nutrition, growth factors, and neuronal activation (Bolshakov et al., 2000; Khoshkhoo et al., 2023) (Figure 2).

In the context of epilepsy, the ERK pathway of MAPK has been shown to stimulate the expression of N-methyl-D-aspartate (NMDA) receptors, which leads to increased synaptic excitability and potentially seizures (Li et al., 2018a). Furthermore, the ERK/MAPK pathway is involved in the induction of mossy fiber sprouting, which is closely associated with epileptogenesis (Nateri et al., 2007). Interestingly, the MAPK pathway responds to different seizure-inducing treatments, suggesting its integral role in epileptogenesis (Pernice et al., 2016).

Another aspect of the MAPK pathway in epilepsy is the misregulation of RNA-binding proteins (RBPs). These proteins play a key role in post-transcriptional gene expression, and the MAPK pathway can influence their activity, which in turn affects the expression of proteins involved in epilepsy (Pernice et al., 2016; Gamarra et al., 2021). Additionally, specific RBPs such as Pumilio-2 have been identified as being involved in epileptogenesis (Follwaczny et al., 2017).

As a result, these altered mechanisms could be implicated in cognitive impairment associated with epilepsy. The p38 MAPK signaling pathway, in particular, modulates apoptosis and impairs cognitive function in rat models of epilepsy (Huang et al., 2017; Asih et al., 2020). This pathway is also associated with refractory epilepsy, where certain inhibitors can reduce seizure frequency and repair damaged hippocampal neurons.

Overall, the link between MAPK activation, LTP, and epilepsy becomes particularly relevant when considering how seizures themselves can induce synaptic changes akin to those seen in LTP. Seizure activity often leads to excessive glutamate release and NMDA receptor overactivation, which can trigger the MAPK pathway. This seizure-induced activation of the MAPK pathway may mimic or enhance the molecular processes involved in LTP, potentially leading to the strengthening of synaptic connections in a maladaptive way that promotes further seizures. Conversely, the MAPK pathway’s role in LTP suggests that modulating this pathway could help in normalizing synaptic plasticity and potentially reduce the hyperexcitability associated with epilepsy.

Overall, the MAPK signaling pathway is a pivotal element in both LTP and epilepsy, establishing a critical link between these two neurological processes (Huang et al., 2017). The activation of the MAPK signaling cascade is fundamental to LTP, it begins with synaptic activation, often initiated by experiential stimuli (Orban et al., 1999). The entry of calcium through NMDA receptors is a crucial step, leading to the activation of the MAPK pathway. This pathway then phosphorylates various intracellular proteins (Li et al., 2019), a process essential for the transcription of genes that are vital for synaptic plasticity and memory consolidation (Yang et al., 2022). Such plasticity enables the strengthening of synapses, thereby facilitating the formation of long-lasting memories (Bohbot and Corkin, 2007).

The MAPK cascade, including the ERK pathway, is involved in hippocampal LTP, demonstrating a direct link between MAPK activation and synaptic plasticity. This connection is important as it underscores the role of the MAPK pathway not just in epilepsy but also in the regulation of synaptic strength and plasticity, which are fundamental to LTP.

In summary, the MAPK pathway plays a critical role in epileptogenesis through its regulation of synaptic excitability and plasticity. Its interaction with RBPs and influence on LTP highlight the pathway’s importance in both the development of epilepsy and the mechanisms underlying synaptic strengthening and learning processes. Understanding the dual role of the MAPK pathway in both promoting physiological synaptic plasticity and contributing to pathological changes in epilepsy underscores its potential as a therapeutic target. Inhibitors of the MAPK pathway may not only dampen the hyperexcitable network characteristics of epilepsy but also help in modulating synaptic plasticity toward a more stable and less excitable state.

## 4 mTOR signaling pathway

The mammalian target of rapamycin (mTOR) pathway is a critical signaling cascade primarily associated with cell growth and proliferation. It influences a range of cellular processes including protein synthesis, transcription, angiogenesis, and autophagy (Wullschleger et al., 2006; Guertin and Sabatini, 2007; Bové et al., 2011). In the nervous system, the mTOR pathway assumes a pivotal role, extending beyond more cell proliferation. It is instrumental in the proliferation of neural stem cells, the assembly and maintenance of neural circuits, and the regulation of complex behaviors (Tang et al., 2014; Figure 1).

In the realm of cognitive neuroscience, the mTOR pathway influences the late LTP, which is crucial for the long-term consolidation of memory. Hyperactivation of the mTOR pathway, as seen in conditions like tuberous sclerosis, leads to abnormal LTP in the hippocampal area CA1. This abnormality manifests as deficits in learning and memory storage, specifically impacting the phase where information is stored rather than the ability to relate information (Joinson et al., 2003; Lee et al., 2012). This indicates that mTOR’s role in LTP is not just in the formation of memory but also in its stabilization and long-term retention.

The mTOR pathway also plays a significant role in epilepsy and epileptogenesis. Abnormal activation of the mTOR pathway contributes to a heightened excitability. In conditions like tuberous sclerosis and epileptic encephalopathy, dysregulation of the mTOR pathway leads to increased cell proliferation and heightened neuronal excitability, mediated by the upregulation of receptors for excitatory neurotransmitters such as glutamate (De Fusco et al., 2020). This increased excitability is a key factor in the development of epilepsy. Moreover, the mTOR pathway can be altered by genetic mutations, brain lesions, or environmental factors, which can act as epigenetic influencers (Blümcke et al., 2011; Epi4K Consortium et al., 2013).

Experimental research has shown that mTOR inhibitors could have therapeutic potential in epilepsy treatment. These inhibitors are being studied for their antiseizure, antiseizure properties (Cho, 2011). The potential for mTOR inhibition in preventing epileptogenesis, particularly in the context of tuberous sclerosis and other common acquired epilepsies, is a significant area of research. Moreover, mTOR inhibitors have shown promise in modulating epileptogenesis, seizures, and even depressive behavior in genetic rat models of absence epilepsy (Russo et al., 2013).

On the other hand, there have been experimental studies exploring the relationship between the mTOR pathway and LTP. One study focused on how ERK regulates the PI3K–mTOR pathway in LTP. It demonstrated that LTP-inducing stimulation increases dendritic translational capacity, involving the convergent effects of PI3K and ERK at the level of PDK1 (Tsokas et al., 2007). Therefore, it supports the idea that the mTOR pathway plays a critical role in the molecular processes underpinning LTP, particularly in terms of protein synthesis and dendritic translational capacity, essential to cognitive processes in the brain (Tsokas et al., 2007; Lee et al., 2012).

The interplay between the mTOR pathway in LTP and epilepsy presents several intriguing questions. Key areas needing further exploration include the mechanisms of mTOR pathway dysregulation in epilepsy, the long-term impact of mTOR inhibitors on cognitive functions, the role of the mTOR pathway in different types of epilepsy, the genetic factors linking mTOR pathway alterations to LTP and epilepsy, the influence of mTOR on other forms of synaptic plasticity like LTD, and comparative analyses of mTOR pathway effects in healthy versus epileptic brain tissues. Addressing these gaps could unveil new therapeutic targets and deepen our understanding of the mTOR pathway’s role in brain functions.

Finally, the mTOR pathway represents a shared mechanism between LTP and epilepsy, primarily through its regulation of neuronal excitability and synaptic plasticity. In the context of LTP, the mTOR pathway is essential for synaptic consolidation and memory formation. Conversely, in epilepsy, dysregulation of this pathway leads to increased neuronal excitability and seizure activity. This dichotomy illustrates the pathway’s dual role in synaptic function: facilitating memory formation and consolidation in a healthy nervous system and contributing to seizure susceptibility and neuronal hyperexcitability in pathological conditions.

mTOR inhibitors, currently used in the treatment of TSC (tuberous sclerosis complex) and other mTOR-related pathologies, have shown promise in reducing seizure frequency and severity by normalizing synaptic protein synthesis and reducing aberrant neuronal growth. These effects also suggest potential benefits in normalizing LTP processes, potentially restoring normal memory and learning mechanisms that are often impaired in patients with epilepsy (Curatolo and Moavero, 2012; Rehbein et al., 2021; Luo et al., 2022).

## 5 Wnt/B-catenin pathway

**FIGURE 3:**
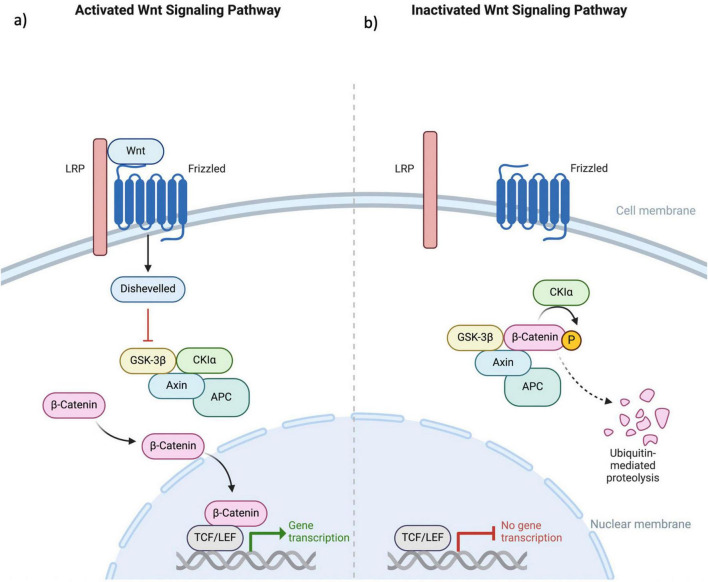
This figure illustrates the **(a)** activated and **(b)** deactivated states within the canonical Wnt signaling pathway. The Wnt protein engages with the extracellular N-terminal region of the Fz receptor family, which consists of a 7-transmembrane domain receptor. Mediation of the Wnt signaling pathway necessitates the involvement of the LRP. In the absence of Wnt coupling to Fz, β-catenin undergoes phosphorylation, marking it for ubiquitin-mediated degradation. This phosphorylation event is specifically mediated by GSK3 within a complex that includes Axin, APC, and CK1α. The binding of Wnt to the receptor results in the downregulation of the Axin, APC, GSK3, and CK1α complex. This inhibition of β-catenin phosphorylation allows its translocation into the cellular nucleus, where it associates with TCF/LEF factors, thereby augmenting the transcriptional activity of various genes. Fz, Frizzled; LRP, low-density lipoprotein receptor-related protein; GSK3, glycogen synthase kinase 3; APC, adenomatous polyposis coli; CK1α, Casein Kinase 1 alpha; TCF/LEF, T-cell factor/lymphoid enhancer factor.

The WNT/β-catenin signaling pathway is fundamental to various physiological processes within the nervous system, including neuronal development, synaptic plasticity, and neurogenesis (Hodges and Lugo, 2018). This pathway involves the binding of WNT proteins to Frizzled receptors, leading to the stabilization of β-catenin in the cytoplasm. Accumulated β-catenin then translocates to the nucleus, where it interacts with TCF/LEF transcription factors to regulate gene expression. This regulation is essential for various aspects of neuronal development, such as differentiation, axonal guidance, and the formation and remodeling of synapses (Freese et al., 2010). The pathway’s role in shaping neural circuitry and influencing synaptic architecture underscores its importance in the overall functionality and health of the nervous system (Figure 3).

In the context of LTP, the WNT/β-catenin pathway plays a crucial role in memory formation and consolidation (Cardamone et al., 2014). The pathway’s influence on synaptic plasticity is particularly significant in the strengthening and formation of synaptic connections, which are key processes in the establishment and maintenance of long-term memory. Activation of WNT signaling can enhance synaptic transmission and facilitate the remodeling of synapses, crucial for the consolidation phase of memory (Bailey et al., 2015). This suggests that the WNT/β-catenin pathway is not only involved in the structural aspects of synaptic connections but also in the functional enhancement of synaptic efficacy, which is a hallmark of LTP.

The WNT/β-catenin pathway has been implicated in the development and progression of epilepsy (Chen et al., 2006). Epileptogenesis involves abnormal neuronal activity and network synchronization. Aberrant activation of the WNT/β-catenin pathway may contribute to this process. Studies have shown that this pathway is upregulated in animal models of epilepsy and in human epileptic brain tissues (Qu et al., 2017; Jean et al., 2022). This upregulation is associated with increased neurogenesis, axonal growth, and synaptic plasticity, potentially leading to the formation of aberrant neural circuits that underlie epileptic seizures. Furthermore, mutations in genes encoding proteins of this pathway can disrupt its balance, leading to altered neuronal function and increased seizure susceptibility (Deisseroth et al., 2004; Ming and Song, 2005).

There are several genes involved in the WNT/β-catenin pathway that have been implicated in epilepsy. Some of these genes are:

1. CTNNB1: This gene encodes β-catenin, a protein that plays a critical role in the WNT/β-catenin pathway. Mutations in CTNNB1 have been associated with focal cortical dysplasia, a common cause of intractable epilepsy (Jessberger et al., 2005; Pecoraro et al., 2017).
2. APC: The adenomatous polyposis coli (APC) gene is a negative regulator of the WNT/β-catenin pathway. Mutations in APC have been linked to familial adenomatous polyposis, a hereditary cancer syndrome that is also associated with an increased risk of epilepsy (Blake et al., 2000).
3. TSC1 and TSC2: TSC is a genetic disorder that can cause epilepsy, among other symptoms. Mutations in the TSC1 and TSC2 genes lead to overactivation of the mammalian target of rapamycin (mTOR) pathway, which can in turn dysregulate the WNT/β-catenin pathway (Mak et al., 2005).
4. GSK-3β: Glycogen synthase kinase-3β (GSK-3β) is a negative regulator of the WNT/β-catenin pathway. Inhibition of GSK-3β activity has been shown to have anticonvulsant effects in animal models of epilepsy (Urbanska et al., 2019).
5. LEF1 and TCF7L2: These genes encode transcription factors that interact with β-catenin to regulate gene expression in the WNT/β-catenin pathway. Polymorphisms in these genes have been associated with an increased risk of epilepsy (Atkinson et al., 2015; Pirone et al., 2017).

It is important to note that the exact roles of these genes in the pathogenesis of epilepsy are not fully understood and may vary depending on the specific type of epilepsy and the underlying genetic and environmental factors involved (Guo et al., 2017).

The WNT/β-catenin pathway represents a shared mechanism between LTP and epilepsy through its regulation of synaptic plasticity and neuronal development. In LTP, the pathway facilitates synaptic strengthening and memory consolidation, essential for long-term memory formation. In epilepsy, however, dysregulated WNT/β-catenin signaling contributes to abnormal neurogenesis and synaptic remodeling, leading to increased neuronal excitability and seizure susceptibility. This shared mechanism highlights the pathway’s dual role in synaptic function: promoting memory formation in a healthy nervous system and contributing to the pathogenesis of epilepsy in a dysregulated state. Several studies have investigated the role of specific WNT/β-catenin pathway components in memory formation and consolidation. For instance, knocking out β-catenin in the hippocampus impairs contextual fear memory in mice, yet it does not lead to changes in seizure development (Zhu et al., 2020; Mercier et al., 2022). Similarly, inhibition of GSK-3β, a negative regulator of the WNT/β-catenin pathway, has been shown to enhance spatial memory in rats (Ge et al., 2020; Mercier et al., 2022).

Other studies have investigated the role of specific WNT/β-catenin target genes in memory formation and consolidation. For example, knockout of the WNT/β-catenin target gene cyclin D1 impairs LTP and spatial memory in mice (Ge et al., 2020; Mercier et al., 2022). Similarly, knockout of the WNT/β-catenin target gene c-myc impairs contextual fear memory in mice.

Furthermore, dysregulation of the WNT/β-catenin pathway has been implicated in the pathophysiology of several neurodegenerative diseases that are associated with memory deficits, including Alzheimer’s disease and Huntington’s disease (Morimoto et al., 1990; Zhu et al., 2020). In Alzheimer’s disease, dysregulation of the WNT/β-catenin pathway has been shown to contribute to the formation of amyloid-β plaques, which are a hallmark of the disease. In Huntington’s disease, dysregulation of the WNT/β-catenin pathway has been implicated in the pathogenesis of the disease (Morimoto et al., 1990).

Modulating the Wnt/β-catenin pathway could normalize the changes in synaptic plasticity and neurogenesis associated with epilepsy. For instance, inhibitors of GSK-3β, a negative regulator of the Wnt pathway, have shown potential in reducing seizure susceptibility and modulating synaptic plasticity (Engel et al., 2018; Jaworski, 2020).

## 6 Ca^2+^ dysregulation

In the epileptic brain, the crux of the matter lies in the aberrant calcium signaling which leads to pathologically enhanced LTP. This enhancement is not merely a quantitative increase in synaptic strength, but qualitatively different from normal LTP, often involving distinct molecular pathways and synaptic changes (Taheri et al., 2023; Romagnolo et al., 2024).

Calcium channels, particularly those affected by genetic mutations, are critical in the pathogenesis of epilepsy. Mutations leading to gain-of-function or loss-of-function in these channels can drastically alter calcium dynamics within neurons. Gain-of-function mutations increase calcium influx, causing elevated intracellular calcium levels, which in turn can excessively activate calcium-dependent pathways involved in LTP. This leads to an aberrant enhancement of synaptic strength, contributing to the hyperexcitable neuronal networks characteristic of epilepsy. On the other hand, loss-of-function mutations decrease calcium influx, altering neuronal excitability and potentially disrupting the normal processes of synaptic plasticity (Hashimotodani et al., 2017; Li et al., 2018b; Figure 1).

For instance, the altered expression or function of specific calcium channels in epilepsy can lead to a sustained and localized increase in intracellular calcium concentration. This can aberrantly activate kinases like CaMKII, overdriving the synaptic potentiation processes that underlie LTP (El Oussini et al., 2023; Taheri et al., 2023).

The implications for memory are profound and complex. In epilepsy, the dysregulated LTP disrupts the selective strengthening of neural pathways. This disruption can lead to “noisy” neural networks where non-essential or even erroneous connections are potentiated, overshadowing or distorting the proper encoding of memories. Moreover, the hyperexcitable networks formed as a result of pathological LTP can interfere with the normal rhythms and patterns of neural activity essential for memory consolidation (Casasola et al., 2004).

In the realm of epilepsy, LTP, and calcium dysregulation, several research gaps persist such as the specific impacts of these disturbances on different types of memory, and the role of genetic variations in calcium channel mutations. Additionally, there’s a need to explore the long-term effects of calcium dysregulation on neural plasticity and memory, develop therapeutic strategies targeting these disturbances to improve cognitive functions in epilepsy and investigate how calcium signaling interacts with other molecular pathways in these contexts. Addressing these gaps could enhance our comprehension and treatment of epilepsy and its cognitive implications.

Therefore, while the fundamental mechanisms of LTP are preserved in epilepsy, their alteration through dysregulated calcium signaling leads to a paradoxical situation. The same mechanism that underpins learning and memory becomes a contributor to cognitive dysfunction, particularly in memory encoding and retrieval. This nuanced understanding is crucial for devising targeted interventions in epilepsy that address not only seizure control but also the preservation and restoration of cognitive functions.

## 7 Conclusion

In conclusion, the exploration of the mechanisms underlying memory and epilepsy, particularly focusing on the hippocampus, highlights a significant overlap in the molecular processes involved in both phenomena. This overlap is evident in the shared molecular mechanisms between kindled seizures and LTP, both induced by similar neural activities. This interrelation underscores the importance of understanding these parallel mechanisms for better therapeutic approaches in epilepsy, aiming not only at seizure control but also at mitigating cognitive dysfunctions, especially in memory encoding and retrieval. The similarities in the pathways governing memory formation and epileptic activity point toward a complex but interlinked neurobiological foundation, critical for developing more effective treatments and understanding the cognitive challenges faced by individuals with epilepsy.
